# Expression of Colonization Factor CS5 of Enterotoxigenic *Escherichia coli* (ETEC) Is Enhanced *In Vivo* and by the Bile Component Na Glycocholate Hydrate

**DOI:** 10.1371/journal.pone.0035827

**Published:** 2012-04-30

**Authors:** Matilda Nicklasson, Åsa Sjöling, Astrid von Mentzer, Firdausi Qadri, Ann-Mari Svennerholm

**Affiliations:** 1 Institute of Biomedicine, Department of Microbiology and Immunology, University of Gothenburg, Göteborg, Sweden; 2 International Centre for Diarrhoeal Disease Research, Dhaka, Bangladesh; University of Osnabrueck, Germany

## Abstract

Enterotoxigenic Escherichia coli (ETEC) is an important cause of acute watery diarrhoea in developing countries. Colonization factors (CFs) on the bacterial surface mediate adhesion to the small intestinal epithelium. Two of the most common CFs worldwide are coli surface antigens 5 and 6 (CS5, CS6). In this study we investigated the expression of CS5 and CS6 *in vivo*, and the effects of bile and sodium bicarbonate, present in the human gut, on the expression of CS5. Five CS5+CS6 ETEC isolates from adult Bangladeshi patients with acute diarrhoea were studied. The level of transcription from the CS5 operon was approximately 100-fold higher than from the CS6 operon in ETEC bacteria recovered directly from diarrhoeal stool without sub-culturing (*in vivo*). The glyco-conjugated primary bile salt sodium glycocholate hydrate (NaGCH) induced phenotypic expression of CS5 in a dose-dependent manner and caused a 100-fold up-regulation of CS5 mRNA levels; this is the first description of NaGCH as an enteropathogenic virulence inducer. The relative transcription levels from the CS5 and CS6 operons in the presence of bile or NaGCH *in vitro* were similar to those *in vivo*. Another bile salt, sodium deoxycholate (NaDC), previously reported to induce enteropathogenic virulence, also induced expression of CS5, whereas sodium bicarbonate did not.

## Introduction

Enterotoxigenic *E. coli* (ETEC) is one of the most common causes of acute watery diarrhoea in the developing world, both among children and adults, causing up to 400 million diarrhoeal episodes and approximately 380.000 deaths in children under the age of five every year. In addition, ETEC causes approximately 400 million diarrhoeal cases among adults. ETEC is also the most common cause of travellerś diarrhoea in these countries [1]–[5]. ETEC is endemic in many countries such as Bangladesh where the infection follows a distinct biannual seasonal pattern with one peak during the hot and dry months of April and May, and a second peak in September and October when the heavy monsoon rains have subsided [6].

ETEC infection is acquired by ingestion of contaminated food or water, and the infection is established when the bacteria reach the small intestine. Diarrhoea is caused by the actions of one or two enterotoxins, the heat-stable (ST) and/or the heat-labile (LT) toxin, which bind to receptors on the epithelial surface, thereby activating signalling pathways that stimulate the opening of ion channels [2], [5], [7] and subsequent release of water and electrolytes into the intestinal lumen. Colonization of the small intestine is mediated by colonization factors (CFs), which are fimbrial, fibrillar or afimbrial structures on the bacterial surface that adhere to the enterocytes. At present at least 25 different CFs have been identified [8]–[11]. Two of the most common CFs expressed by clinical isolates from different parts of the world are the coli surface antigens 5 (CS5) and 6 (CS6) [5], [9], [12]–[14]. CS5 and CS6 belong to the colonization factor antigen group IV (CFA/IV) which also contains coli surface antigen 4 (CS4). CS5 is always expressed together with CS6, while CS6 can also be expressed alone or together with CS4 [9], [15]. CS5 is a fibrillar antigen, and its operon encodes a major subunit (*csfA*), a minor subunit (*csfD*), an outer membrane usher (*csfC*), two chaperones (*csfB* and *csfF*), and a protein involved in pilus length regulation (*csfE*) [16]. CS6 is afimbrial and is composed of two structural subunits, CssA and CssB, located on the bacterial cell membrane. The CS6 operon contains four genes, *cssA*, *cssB*, *cssC*, and *cssD*, encoding the two structural subunits CssA and CssB, a chaperone (CssC), and an usher (CssD), respectively [17]. The current knowledge about the regulation of CS5 and CS6 is limited, and the present study was conducted in order to increase the understanding of the regulation of these important virulence factors in the human gut, with an emphasis on CS5.

When bacteria enter and colonize the gastrointestinal tract they encounter a variety of host environmental factors that may influence virulence gene expression, e.g. bile and sodium bicarbonate, which have previously been shown to regulate expression of virulence factors in other enteropathogens [18]–[21]. NaHCO_3_ is secreted from the pancreas into the duodenum, and is also released from the CFTR channels which results in an increase in pH. Bile has detergenic properties and acts as an emulsifier of dietary fats and lipids *in vivo* [22] and plays a role in antimicrobial defence [23]. It is produced by the liver and contains many components including bile acids, cholesterol, phospholipids, and biliverdin [23]. Primary bile acids are produced directly from cholesterol in the liver by the hepatocytes, and secondary bile acids are produced by bacteria in the caecum and colon by modifications of the primary bile acids. Primary and secondary bile acids are modified by the hepatocytes by conjugation with either glycine (glyco-conjugated bile acids) or taurine (tauro-conjugated bile acids) [22], [23]. Bile salts are secreted into the duodenum at estimated concentrations of 0.2–2% [24].

In this study we investigated the expression of CS5 and CS6 in ETEC bacteria isolated without sub-culturing from clinical stool samples (*in vivo*). The effects of sodium bicarbonate and bile on the expression of CS5 were investigated in *in vitro* cultured bacteria. A certain component of bile, sodium glycocholate hydrate, was identified as a potent inducer of CS5 expression. To our knowledge this is the first report that implicates this molecule as an inducer of enteropathogenic bacterial virulence.

## Results

### CS5+CS6, LT+ST ETEC Strains Isolated from Bangladeshi Stool Specimens

Four clinical ETEC strains from stool specimens collected in 2005 and 2006 from adult patients with acute diarrhoea at the icddr,b hospital in Dhaka were included in the study (Table 1). An LT+STh, CS5+CS6 laboratory reference strain (E3003; sometimes referred to as VM 75689 in other studies), originally isolated in Dhaka in 1988, was also included. A total of 127 clinical stool specimens were collected in 2005 between April and June during the ETEC peak season. Out of a total of 34 identified ETEC positive stool specimens, five contained a CS5+CS6 positive strain that expressed both the ST and the LT toxin. Strains E1777, E1779, and E1785 were chosen for subsequent studies. A co-infecting pathogen was only detected for the patient infected with strain E1779, who had a co-infection with *V. cholerae* Ogawa.

**Table 1:** Strains used in the study and results from culturing^a^ of clinical stool specimens.

ETECstrain^b^:	Culture onMacConkeyagar *^c^*	Culture onTTGA agar	CF and toxin profileof isolated ETEC strain	Year ofisolationin Dhaka
**E1777**	*E. coli*	negative	CS5+CS6, STh+LT	April 2005
**E1779**	*E. coli*	*V.cholerae* Ogawa	CS5+CS6, STh+LT	May 2005
**E1785**	*E. coli*	negative	CS5+CS6, STh+LT	June 2005
**E2265**	*E. coli*	negative	CS5+CS6, STh+LT	March 2006
**E3003** ***^c^***	*E. coli*	N.T.	CS5+CS6, STh+LT	1988

a Culture was performed on MacConkey agar plates to detect *E. coli* and on Taurocholate-tellurite-gelatin agar (TTGA) for detection of vibrios; all cultures were performed overnight at 37°C.

b Department of Microbiology and Immunology, University of Gothenburg, enterotoxigenic *Escherichia coli* (ETEC) strain collection number.

### Immune Responses Against the CS5 and CS6 ETEC Strains

**Figure 1:**
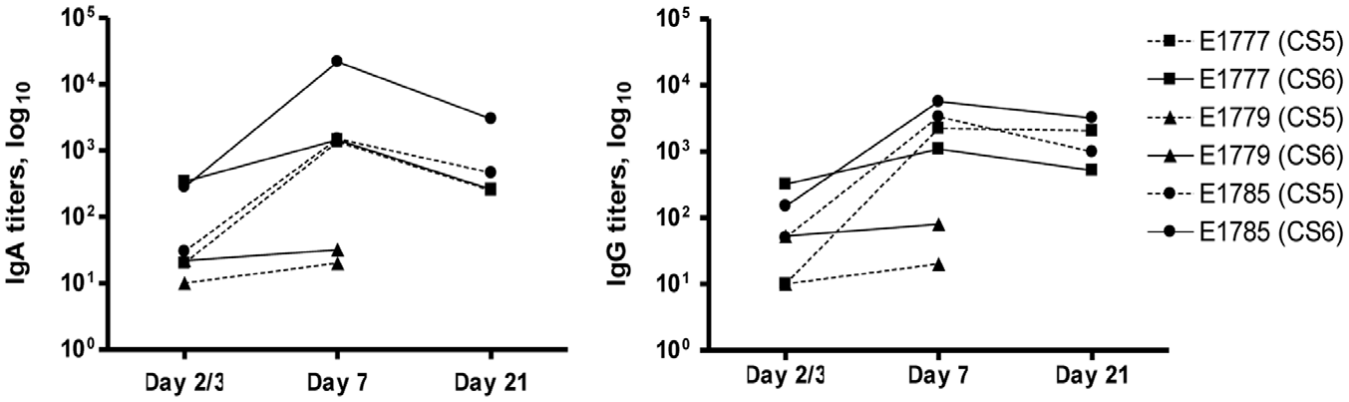
IgA and IgG serum antibody responses to CS5 and CS6 in individual patients. The responses to CS5 (broken lines) and CS6 (solid lines) were measured by ELISA in sera from patients infected with strains E1777, E1785 and E1779, on different days after hospitalization. Serum from the patient infected with strain E2265 was not available.

ELISA analyses of serum samples from the three patients from whom the CS5+CS6 positive strains E1777, E1779, and E1785 had been isolated showed that on day 7 (early convalescence) the patients infected with E1777 and E1785 had developed strong IgG as well as IgA immune responses against CS5, whereas the patient infected with strain E1779, who had a co-infection with *V. cholerae* Ogawa, had developed only a very modest immune response (IgA and IgG) against CS5 (Fig. 1). The patient infected with strain E1785 also developed a strong immune response (IgA and IgG) against CS6, whereas those infected with strains E1777 and E1779 exhibited modest immune responses (Fig 1). These results confirm that CS5 and CS6 were indeed expressed and exposed on the bacteria during the infection. The IgA responses induced against CS6 were comparable to those previously reported for Bangladeshi adults infected with CS5+CS6 ETEC strains [25].

### Transcription of the CS5 and CS6 Operons *in vivo*

**Figure 2:**
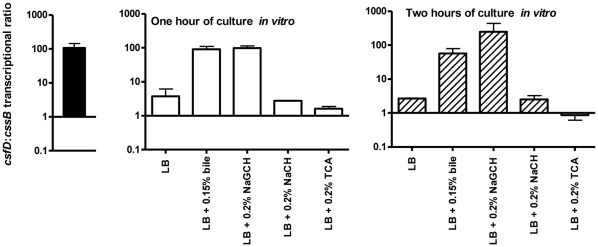
Transcriptional *csfD*:*ccsB* ratio *in vivo* and *in vitro*. Transcription of *csfD* and *cssB* was quantified by real time reverse transcriptase PCR (rt RT-PCR) in *in vivo* bacterial samples (strains E1777, E1785, E2265; closed bar) and after one (strains E1785, E2265, E3003; open bars) and two (E1777, E1779; hatched bars) hours of *in vitro* culture in LB medium alone and in LB supplemented with 0.15% crude bile or 0.2% individual bile salts. Bars show the means and standard errors of the means of two (after two hours of culture) or three (*in vivo* and after one hour of culture) separate experiments.

The transcription levels of the CS5 and CS6 operons in *in vivo* bacterial samples was analyzed by extracting total RNA directly from diarrhoeal stool bacteria and measuring the transcription levels of the structural genes *csfD* and *cssB* of CS5 and CS6, respectively, by quantitative real time reverse transcriptase PCR (rt RT-PCR). Since the *E. coli* bacterial load in the diarrhoeal stool specimens was unknown, and since there are no reliable *E. coli* housekeeping genes for relative quantification, the relative ratio of the transcription levels of *csfD* and *cssB* was determined and compared instead of analyzing the transcription level of each individual gene in relation to a housekeeping gene or a fixed amount of RNA (Fig. 2). Transcription of the *E. coli* housekeeping gene *gapA* was detected in all samples, concluding that intact *E. coli* mRNA was present in all *in vivo* bacterial samples. Transcription of *csfD* and *cssB* was observed in the three *in vivo* bacterial samples in which ETEC was the only detected bacterial pathogen, *i.e.* those containing strains E1777, E1785 and E2265 (Table 1). The sample that contained *V. cholerae* in addition to ETEC, i.e. strain E1779, was negative for transcription of both *csfD* and *cssB*; since transcription of *gapA* was present, this was not due to the deterioration of RNA but indicative of low levels of ETEC bacteria in this particular stool sample.

### Bile, but not Sodium Bicarbonate, Induces Phenotypic Expression of CS5 both under Nutrient-rich and Nutrient-poor Conditions

In order to investigate whether the expression of CS5 by ETEC bacteria *in vivo* may be regulated by the host environmental factors bile and NaHCO_3_, which are both abundant in the human intestine, the strains were analyzed by an inhibition ELISA after *in vitro* culture in the presence and absence of these factors. After overnight culture to stationary phase in LB medium (all five strains) as well as in M9 minimal medium supplemented with glucose, glycerol, or casamino acids as the primary carbon source (strains E1779 and E2265), CS5 was only detected on the bacterial surface when bile was present in the culture medium. Crude bile is routinely added to the culture medium (CFA medium) under standard diagnostic laboratory conditions in order to enable detection of phenotypic CS5, e.g. by dot-blot analysis [26]. Our results indicate that the presence of bile is required for phenotypic CS5 expression not only under nutrient-rich conditions, but also under nutrient-poor conditions. NaHCO_3_ was tested at concentrations 0.3% (35.7 mM) and 0.6% (71.4 mM), which fall within the concentration range previously reported to positively regulate the expression of virulence factors in other enteropathogenic bacteria, i.e. *Vibrio cholerae* and enterohemorrhagic *E. coli* (EHEC) [19], [20]. However, overnight culture to stationary phase in LB medium supplemented with NaHCO_3_ did not result in phenotypic expression of CS5 in any of the five strains included in the study, indicating that this host factor is not an inducer of CS5 expression.

### Sodium Glycocholate Hydrate Induces Phenotypic Expression of CS5

**Figure 3:**
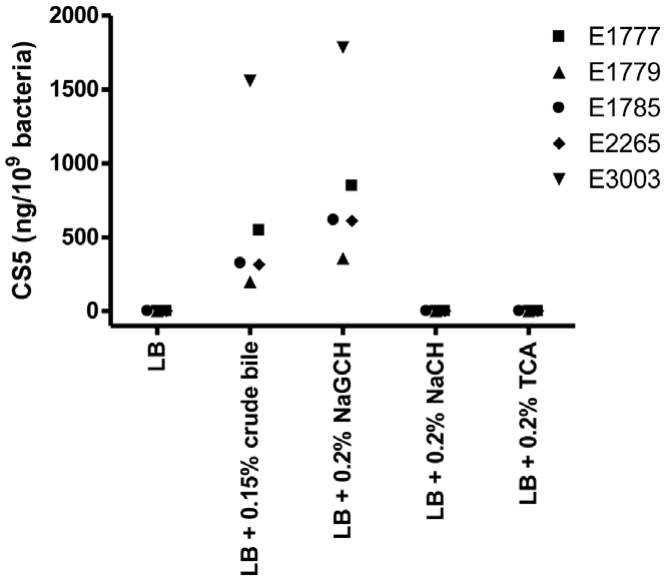
Phenotypic CS5 levels in LB alone and LB supplemented with bile or individual bile salts. CS5 expression was quantified by inhibition ELISA after overnight culture to stationary phase of strains E1777, E1779, E1785, E2265, and E3003. CS5 surface expression tifwas induced by NaGCH, but not by the corresponding unconjugated bile salt NaCH or its tauro-conjugated counterpart TCA (representative data).

The induction of CS5 expression by crude bile was found not to be due to non-specific induction by the detergenic properties of bile, since overnight culture to stationary phase in LB supplemented with Triton X-100 (0.1% and 0.04%) did not result in phenotypic CS5 expression in any of the five strains included in the study. Approximately 50% of the organic compounds in bile are constituted by bile acids [23]. To individually investigate the effects of some of the major bile salts in human bile on CS5 expression, strain E1777 was cultured to stationary phase in LB supplemented with the primary bile salts, i.e. sodium cholate hydrate (NaCH) and sodium chenodeoxycholate (NaCDC) [23], respectively; the glyco- and tauro-conjugated salts of NaCH, i.e. sodium glycocholate hydrate (NaGCH) and taurocholic acid sodium salt hydrate (TCA), respectively; and the secondary bile salt sodium deoxycholate (NaDC), which is derived from cholic acid by dehydroxylation [23]. The individual bile salts were tested at final concentrations of 0.04% and 0.2% (NaCH), 0.04%, 0.1% and 0.2% (NaDC), 0.04%, 0.1% and 0.2% (TCA), 0.04% (NaCDC), and 0.04%, 0.1% and 0.2% (NaGCH). Expression of CS5 on the bacterial surface was induced by NaDC (results not shown), which is known to induce virulence in other enteropathogenic bacteria [21], [27]–[30], as well as by the glycoconjugate NaGCH. The tauro-conjugated counterpart of NaGCH, TCA, and the corresponding unconjugated bile salt NaCH, did not induce phenotypic CS5 expression, irrespectively of the concentration used. The ability of NaGCH and the inability of NaCH and TCA to induce CS5 surface expression was confirmed for the clinical strains E1779, E1785, and E2265 as well as the reference strain E3003, using a concentration of 0.2% of each individual salt (Fig. 3).

**Figure 4:**
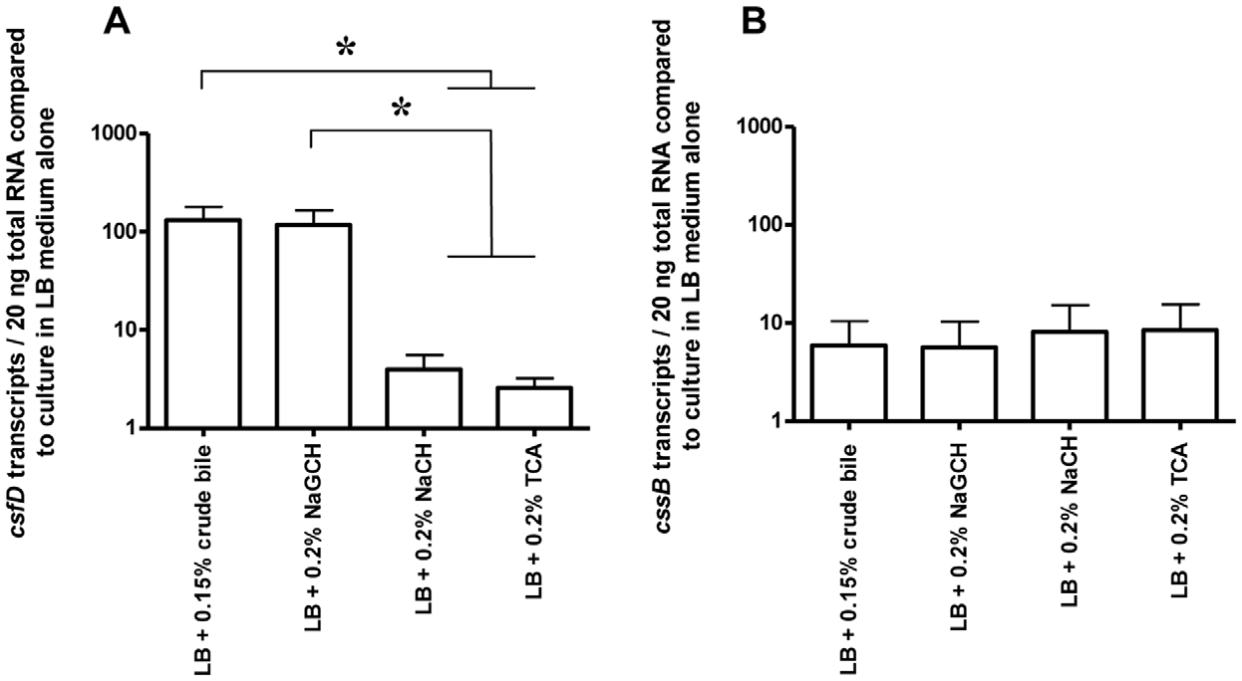
Levels of *csfD* and *cssB* transcription compared to in LB alone after one hour of culture. Level of *csfD* (A) and *cssB* (B) transcription in LB supplemented with crude bile or individual bile salts standardized to the level of transcription in LB medium alone, after one hour of culture. Transcription was measured by reverse transcriptase real time PCR. Bars show means and standard errors of the means of three separate experiments (strains E1785, E2265, and E3003, respectively). *, P = <0.05

### Transcription of *csfD* is Induced by Bile and NaGCH *in vitro*

The novel discovery that NaGCH may act as an inducer of virulence was further investigated by studying the effects on transcription of the CS5 operon by NaGCH, TCA, and NaCH. As comparison, the effects of crude bile were also investigated. This was performed by quantification of the transcription of *csfD*, which encodes the CS5 minor structural subunit, in strains cultured to exponential growth phase in LB medium alone and in LB medium supplemented with 0.15% crude bile or 0.2% NaGCH, 0.2% NaCH or 0.2% TCA, respectively. The transcription of *csfD* was up-regulated approximately 100-fold after one hour of culture when LB was supplemented with 0.15% crude bile or 0.2% NaGCH as compared to after culture in LB alone (Fig. 4). The up-regulation of *csfD* transcription was significantly higher in LB medium supplemented with 0.15% crude bile or 0.2% NaGCH, respectively, than in LB medium supplemented with 0.2% NaCH or 0.2% TCA after one (Fig. 4), two and five hours of culture (data not shown). Transcription of the CS6 structural subunit gene *cssB* was up-regulated approximately 10-fold after the first hour of culture in LB medium supplemented with 0.15% crude bile or 0.2% NaGCH as compared to in LB medium alone (Fig. 4), and less than 10-fold up-regulated or even down-regulated after two and five hours of culture (not shown), indicating that the up-regulation of *csfD* in the presence of bile or NaGCH was specific for the CS5 operon and was not due to a general up-regulation of transcription.

### The Induction of CS5 Expression by NaGCH is Dose-dependent

**Figure 5:**
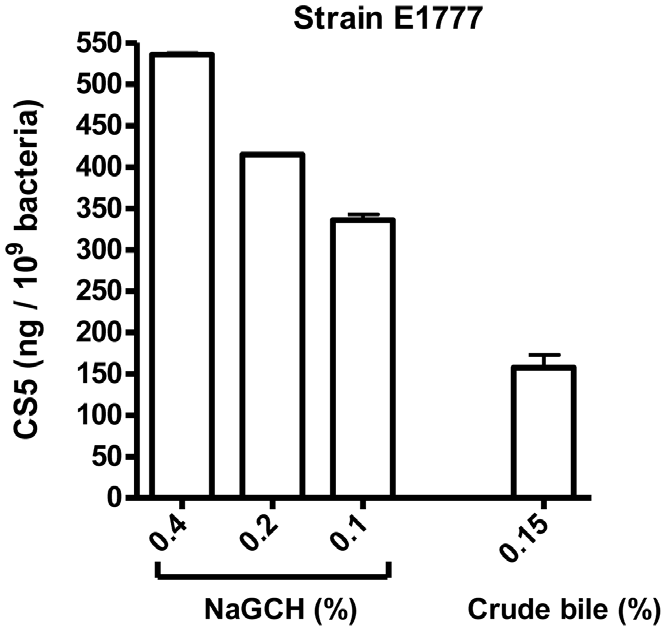
Dose-dependent induction of phenotypic CS5 expression by NaGCH. Expression of CS5 was determined by inhibition ELISA in strain E1777 after overnight culture to stationary phase in LB medium supplemented with NaGCH or crude bile. Bars indicate means and standard errors of the means of two measurements in one experiment.

Inhibition ELISA analyses of strain E1777 after overnight culture to stationary phase in LB supplemented with NaGCH to a final concentration of 0.4%, 0.2%, and 0.1%, respectively, revealed that the induction of phenotypic CS5 expression by NaGCH is dose-dependent (Fig. 5). Phenotypic expression of CS5 was also detected when a NaGCH concentration of 0.04% was tested, but a NaGCH concentration of 0.004% was not sufficient for induction of phenotypic CS5 expression (data not shown). The induction of CS5 expression by crude bile is shown as comparison in Fig. 5.

### The Ratio Between *csfD* and *cssB* Transcription Levels is Highest *in vivo* and in the Presence of Bile or NaGCH *in vitro*

*In vivo*, i.e. in bacteria obtained directly from the stool specimens without sub-culturing, the level of transcription of *csfD* was higher than *cssB*. The transcriptional ratios of *csfD* to *cssB* (*csfD*:*cssB*) were found to be 182∶1, 65∶1, and 76∶1 for the samples containing strains E1777, E1785, and E2265, respectively. Similar *csfD*:*cssB* ratios were observed *in vitro* after one and two hours of culture in LB medium supplemented with 0.15% crude bile or 0.2% NaGCH (Fig. 2). On the other hand, culture in LB medium alone or supplemented with 0.2% TCA or 0.2% NaCH did not result in a similar ratio between *csfD* and *cssB* transcription. Furthermore, the relative levels of transcription of *csfD* and *cssB* in the presence of NaGCH was found to be dose-dependent, as determined for strain E1777; the obtained *csfD*:*cssB* ratios were 2∶1 after overnight culture to stationary phase in LB supplemented with 0.004% NaGCH, 75∶1 in 0.04% NaGCH, 151∶1 in 0.1%, 163∶1 in 0.2%, and 207∶1 in 0.4% NaGCH, which supports that NaGCH is a specific inducer of transcription of the CS5 operon.

## Discussion

Diarrhoea caused by ETEC seems to require colonization of the human intestine by different colonization factors. In this study we have investigated the expression of the important ETEC colonization factors CS5 and CS6 *in vivo*, as well as the role of certain host factors in the human gastrointestinal tract as putative regulators of CS5. These analyses have shown that expression of CS5 is strongly enhanced *in vivo* as compared to CS6 and that a specific factor in the human intestine, i.e. the glyco-conjugated primary bile salt sodium glycocholate (NaGCH) can act as a positive regulator of CS5 expression. To our knowledge, this is the first description of induction of virulence by this bile component.

Relatively little has previously been known about the regulation of CS5, especially *in vivo*. Under standard laboratory conditions (overnight culture on CFA medium at 37°C), crude bile is routinely added to the culture medium in order to enable phenotypic expression of CS5 [26]. Our results show that bile induces expression of CS5 not only in nutrient-rich media, but also under nutrient-poor conditions where only amino acids, glycerol, or glucose constitute the carbon source. In this study we wanted to further investigate the dependency on bile for phenotypic CS5 expression to occur, and to elucidate how this induction comes about. The induction was first determined not to be due to non-specific effects of the detergent properties of bile, since addition of a detergent (Triton X-100) to the culture medium failed to induce phenotypic CS5 expression. Since bile salts form a major constituent of human bile [23], we next decided to test some of the major bile salts in human bile for effects on CS5 expression. A number of enteric bacterial pathogens have previously been reported to switch on the expression of virulence factors in the presence of bile salts. For instance, in atypical enteropathogenic *Escherichia coli* (aEPEC), phenotypic expression of the major subunit of the afimbrial LDA adhesin has been reported to be enhanced by a mixture of sodium deoxycholate, sodium taurodeoxycholate, and sodium chenodeoxycholate [30]. In *Campylobacter jejuni*, sodium deoxycholate, sodium cholate and sodium chenodeoxycholate stimulate the production of *Campylobacter* invasion antigens (Cia), thus affecting the invasiveness of the bacteria [29], and in *V. cholerae* sodium cholate, sodium deoxycholate and sodium taurocholate have been found to induce phenotypic expression of the cholera toxin under certain *in vitro* conditions [21]. In the case of ETEC, individual bile salts have not previously been investigated for their effects on the expression of the colonization factors. In this study we show that one of the bile salts previously known to induce the expression of virulence factors in *Campylobacter, Shigella*, *Salmonella*, and *V. cholerae*, sodium deoxycholate (NaDC), also induces the expression of CS5. More importantly, we were able to identify another positive regulator of the common CS5, the glyco-conjugated primary bile acid sodium glycocholate (NaGCH, or glycocholic acid in its acid form). To the best of our knowledge this is the first study to report that this bile acid can serve as an inducer of bacterial virulence. The crude bile extract (Oxoid) that is routinely used for diagnostic purposes for detection of CS5 is mainly composed of NaGCH and the sodium salt of the tauro-conjugated counterpart of NaGCH, sodium taurocholate (TCA), the latter of which we found is not able to induce expression of CS5. Since glycocholic and taurocholic acid have also been reported to be the main constituents of bile [31], one may speculate that NaGCH is more important for CS5-mediated virulence than NaDC *in vivo*. Glycocholate is formed by the linking of a glycine amino group by an amide bond to the carboxyl group of the primary bile acid cholic acid [23]. Considering that sodium cholate (NaCH) and TCA did not induce expression of CS5, this indicates that the glycine amino group in NaGCH is somehow a prerequisite for NaGCH to positively regulate virulence gene expression. Studies to further elucidate the role of the glycine group have been initiated, as well as to determine the molecular basis of the positive regulation of CS5 expression by NaGCH.

The induction of CS5 on the bacterial surface by NaGCH was coupled with a dose-dependent up-regulation of the ratio between *csfD* and *cssB* transcription levels, suggesting that regulation of CS5 expression at least partly occurs on the transcriptional level. Interestingly, NaGCH has previously been reported to activate certain promoters involved in *E. coli* stress responses [32]. While the CS5 operon itself does not encode a specific regulatory protein, the AraC/XylS-like transcription factor CsvR has been suggested to positively regulate CS5 transcription [33], [34]. These, and other possible pathways of regulation of CS5 expression by NaGCH, are being explored further. In contrast to CS5, no positive regulator has yet been demonstrated for CS6 [35]. The transcription of the CS6 operon was not up-regulated to the same extent by NaGCH as the transcription of the CS5 operon, suggesting that these two operons are under the control of different regulators.

In the *in vivo* bacterial samples (collected from diarrhoeal stool without sub-culturing), we found that the CS5 operon was transcribed at approximately 100-fold higher levels than the CS6 operon. Interestingly, these levels were similar to the levels of up-regulation which were observed *in vitro* after one and two hours of culture, when crude bile or NaGCH was added to the culture media. Taken together, these results suggest that the conditions encountered by ETEC bacteria in diarrhoeal stool are mimicked by the conditions prevailing after one hour of culture in LB medium supplemented by 0.2% NaGCH or 0.15% bile, at least when it comes to transcription of the CS5 and CS6 operons.

Transcription of *csfD* and *cssB* was detected in three of the four patients included in the study; the exception was a patient who had a co-infection with *V. cholerae*. This patient also exhibited a very modest immune response to CS5 and CS6. Hence, it is tempting to speculate about whether possible interactions between ETEC and a co-infecting enteropathogen such as *V. cholerae* may result in reduced expression of ETEC virulence genes, e.g. CS5. However, further studies are needed to explore this.

In the *in vivo* setting, bile salts are secreted into the duodenum at estimated concentrations of 0.2–2% [24]. In this study most individual bile salts were tested at a concentration of 0.2%, since a concentration of 0.15% crude bile is sufficient to induce expression of CS5. However, the concentration of individual bile salts *in vivo* is affected by the nutritional status of the host, e.g. the ingestion of a fatty meal will increase their concentration, and the concentration of bile in the intestinal lumen is lower in malnourished patients [23]. The ratio of glyco-conjugated (e.g. NaGCH) to tauro-conjugated (e.g. TCA) bile acids has also been reported to vary. It has been reported to be 3∶1 in the normal case, but to be much lower (0.1∶1) in taurine-fed individuals. Foods that are rich in taurine include meat and sea-food, which will increase tauroconjugation [23]. In the light of our findings, this suggests that the diet of an individual may influence the outcome of an infection with CS5 positive ETEC, since certain diets may decrease the ratio between glyco- and tauro-conjugates. One may speculate whether a diet lacking adequate levels of taurine, e.g. lacking in meat, which is a common occurrence in poor socioeconomic settings where ETEC infections are common, may further increase the severity of CS5 ETEC infections. The composition of bile also varies between different populations, eg. in rural African women the ratio of glyco- to tauro-conjugated bile acids may be as high as 9∶1 [23].

Our results showed that the induction of CS5 is dose-dependent; a final concentration of sodium glycocholate of 0.004% was not enough to induce phenotypic expression, but a gradual increase in induction was observed when increasing the concentration of the salt from 0.04% to 0.4%. Bile is delivered from the gall bladder into the proximal part of the small intestine and sequentially decreases in concentration along the intestine due to re-absorption to the liver. In addition, the bile level is likely to differ between the intestinal lumen and the mucosal layer. Our findings suggest that CS5 ETEC bacteria are capable of fine-tuning the expression of CS5 according to the concentration and composition of bile, in order for optimal expression to occur at different sites and under varying nutritional conditions in the human intestine. CS5 is only expressed in ETEC strains that are also capable of expressing CS6 [36]. Unlike CS5, however, CS6 is not dependent on the presence of bile in order to be expressed *in vitro*, which suggests that expression of these two important colonization factors may be induced by different environmental niches within the host.

Another abundant host factor in the human small intestine, NaHCO_3_, was also tested but ruled out as a putative regulator of CS5. The concentration of NaHCO_3_ in the intestine is difficult to measure and exhibits great variability. The concentrations used by us, 35.7 mM and 71.4 mM, fall within the concentration range previously reported to positively regulate the virulence factors in other enteropathogens, i.e. *V. cholerae* and enterohemorrhagic *E. coli* (EHEC). Thus, 35.7 mM NaHCO_3_ has been found to upregulate CT and TCP in *V. cholerae* [19], and 44 mM to induce the production of virulence factors in EHEC [20].

In summary, we report the novel findings that the ETEC colonization factor CS5 is considerably more expressed *in vivo* than CS6 when co-expressed on the same bacteria and that the bile component sodium glycocholate, which is abundant in the human intestine, induces phenotypic expression of CS5 in a dose-dependent manner.

## Materials and Methods

### Ethics Statement

This study was conducted according to the principles expressed in the declaration of Helsinki. The study was approved by the Research Review Committee and the Ethical Review Committee of the International Centre for Diarrhoeal Disease Research, Bangladesh (icddr,b) (protocol# 2001-027). Ethical permission for the study was also obtained from the Regional Ethical Review Board, Gothenburg, Sweden (S199-03 and 088-10). Written informed consent was obtained from study participants prior to enrollment in the study and the collection of stool and blood specimens. No children were included in the study.

### Collection of *in vivo* Bacterial Samples from Stools

Watery diarrhoeal stool specimens were collected from adult patients with acute diarrhoea admitted to the hospital of the International Centre for Diarrhoeal Disease Research (icddr,b) in Dhaka, Bangladesh. Fresh stool specimens (10–50 ml) were placed on ice and brought from the hospital wards to the laboratory where bacteria present in the stool samples were immediately isolated essentially as previously described [37] with some modifications. The stool samples were filtered through sterilized gauze and centrifuged at 1000×g for 10 minutes in order to remove mucus and other large particles. The supernatant, containing the bacteria, was carefully removed and centrifuged at 16000×g for 10 minutes. The resulting bacterial pellet, *i.e.* the *in vivo* bacterial sample, was resuspended in 1 ml RNALater® (Qiagen, Hilden, Germany), divided into two aliquots of 500 µl, stored at -70°C and shipped to Sweden for total RNA extraction and quantification of transcription.

### Characterization of Stool Specimens and Clinical ETEC Isolates

Bacteriological analysis of the stool specimens was performed essentially as previously described [14]. Specimens were initially screened for *V. cholerae* by dark-field microscopy; negative specimens were further screened for presence of *E. coli-*like bacteria, and *V. cholerae*, *Shigella* spp, and *Salmonella* spp by standard methods [14], [38]. Six lactose-fermenting *E. coli*-like colonies identified after culture on MacConkey medium were individually tested for ST and LT by ELISA assays and for the CFs CFA/I, CS1-8, CS12, CS14, CS17 and CS21 by dot-blot assays [26], [39], [40]. DNA from a pool of the six colonies was tested for the genes encoding LT, STh, and STp by PCR [26], [41]. One representative ETEC colony from each ETEC positive stool specimen was grown to exponential phase in LB medium and stored at -70°C in LB supplemented with 20% glycerol; these strains were further used in the *in vitro* experiments.

### Collection of Blood and Serum Specimens

Blood samples were collected from the patients at the acute stage of the infection, *i.e.* on day 2 or 3 of hospitalization, and during convalescence on days 7 (early convalescence) and 21 (late convalescence), and stored at -20°C until analyses.

### *In vitro* Culture Conditions

The ETEC isolates were cultured overnight on LB or blood agar plates at 37°C. Three or six colonies were picked and grown to exponential phase in LB (pH 7.5) at 37°C with aerated shaking (150 or 200 rpm) to reach exponential phase. This starting culture was inoculated into the media to be tested at a final concentration of 10^7^ or 10^8^ bacteria/ml, based on the absorbance at OD_600_, and cultured overnight at 37°C with shaking (150 rpm). Media tested were LB, CFA broth [42], and defined minimal medium (M9). M9 medium was supplemented with either glucose (0.2% w/v), glycerol (0.2% w/v), or casamino acids (1% w/v) as the primary carbon source. When relevant, crude bile (Oxoid, Hampshire, UK) was added to a final concentration of 0.15% w/v [26], or 0.015% (in the case of M9). LB medium was supplemented with Triton X-100 (Sigma, St Louis, MO), sodium bicarbonate (Sigma), or individual bile salts (Sigma), i.e. sodium cholate hydrate (NaCH), sodium deoxycholate (NaDC), taurocholic acid sodium salt hydrate (TCA), sodium chenodeoxycholate (NaCDC), and sodium glycocholate hydrate (NaGCH), respectively, at different concentrations as indicated.

### Enzyme-linked Immunosorbent Assay (ELISA)

The phenotypic expression of CS5 and CS6 on the bacterial surface was quantified by inhibition ELISA assays after overnight culture to stationary phase, essentially as described [43], with a starting concentration of 10^9^ or 10^10^ bacteria/ml.

### Total RNA Extraction from *in vitro* Bacterial Cultures

The pellet from one ml of bacterial culture was treated with lysozyme and RLT buffer (Qiagen) and stored at -70°C. Total RNA was prepared from a volume corresponding to 10^9^ lysed bacteria with the RNeasy^®^ Mini Kit (Qiagen, Hilden, Germany), removing contaminating DNA on-column by using the RNase-Free DNase Set (Qiagen). Alternatively, approximately 10^9^ bacteria were spun down, treated with RNAProtect® (Qiagen) and stored at -70°C before total RNA extraction. The integrity of the RNA and absence of contaminating DNA was checked by agarose gel electrophoresis and the RNA concentration was measured spectrophotometrically using a NanoDrop^®^ ND-1000 (NanoDrop Technologies, Wilmington, DE). All RNA samples were stored at -70°C.

### Total RNA Extraction from *in vivo* Bacterial Samples

*In vivo* bacterial samples in RNALater were thawed on ice and spun down at 4°C. The pellet was treated with an appropriate volume of lysozyme and RLT buffer (Qiagen) before total RNA extraction using the RNeasy® Mini Kit (Qiagen). Contaminating DNA was removed by twice performing the on-column DNase treatment described above.

### cDNA Synthesis

cDNA was synthesized from a maximum of 200 ng total RNA using the QuantiTect Reverse Transcription Kit (Qiagen), which includes an additional DNAse treatment step. Controls for DNA contamination without reverse transcriptase (-RT) were prepared simultaneously with the synthesized cDNA from the same amount of total RNA. cDNA and (-RT) controls were stored at -20°C.

### Quantitative Real Time RT-PCR Assays

The real-time RT-PCR assays were performed on an ABI 7500 (Applied Biosystems, Foster City, CA) using the double-stranded DNA-specific dye SYBR®Green I (Applied Biosystems, Warrington, UK) as detector. Primers for the CS5 minor structural subunit gene *csfD* and the *E. coli* housekeeping gene *gapA* were designed using the Primer Express 2.0 software (Applied Biosystems, Foster City, CA). The primers for the CS6 structural subunit gene *cssB* have been described previously [44] (Table 2).

**Table 2:** Primers used for PCR and real-time Reverse Transcriptase PCR (rt RT-PCR).

Gene	Primers (5′→3′)	Direction	Product size
*csfD* (CS5) a	ACTTGGATATTGGTCGACTGCAA	Forward	101 bp
	ATTCTTTAAAACCTCTCCAGCGAAT	Reverse	
*cssB* (CS6) b, c	CAGGAACTTCCGGAGTGGTA	Forward	152 bp
	CTGTGAATCCAGTTTCGGGT	Reverse	
*gapA* d	CGTTGAAGTGAAAGACGGTCATC	Forward	101 bp
	CAACACCAACTTCGTCCCATTT	Reverse	

a CS5 minor subunit**;** GenBank accession number AJ224079.

b CS6 structural subunit CssB; GenBank accession number UO4846.

c Primers described in [44].

d *E. coli* D-glyceraldehyde-3-phosphate dehydrogenase (GAPDH); GenBank accession number U014639.1.

The transcription levels were determined by absolute quantification using a standard curve for each gene which was generated by ten-fold serial dilutions of a known amount of specific PCR product. These PCR products were generated by conventional PCR from DNA isolated from laboratory strains (E17018/A for *csfD*; E11881/14 for *cssB* and *gapA*), using the same primers as for real-time PCR. PCR products were purified by the QIAQuick PCR Purification Kit (Qiagen) or the E.Z.N.A Cycle Pure Kit (Omega Bio-Tek, Inc., Norcross, GA), and their concentrations were carefully determined on a NanoDrop^®^ ND-1000 (NanoDrop). The number of PCR products per µl was calculated as previously described [37]. The PCR products were then diluted in Elution Buffer (Qiagen, Omega Bio-Tek) to give ten-fold dilution series from 5×10^7^ to 5 PCR products/µl.

All real-time PCR reactions were performed in 20 µl volumes containing 8 pmol of each specific primer, 10 µl Power SYBR®Green PCR Master Mix (Applied Biosystems), and 2 µl of template, using the default cycling settings with minor modifications as described in [45]. Quantification was performed by using the default software settings for the threshold and baseline values (Applied Biosystems). The number of gene-specific transcripts per 20 ng of total RNA was calculated. cDNA samples and corresponding (–RT) controls were run in parallel. When the difference in C_t_ values between the sample and the (–RT) control was ≥ 3.0 or ≤ 6.6, the number of transcripts was adjusted accordingly. When the difference was > 6.6 the (–RT) values were ignored since any contaminating DNA would account for less than 1% of the total product, but if the difference was < 3.0 the amount of DNA contamination was considered too high and the results were discarded. Since we have previously found a detection limit of 2–3 copies per reaction for real-time PCR [45] the detection limit in this study was set at 2 transcripts per reaction, and all results below 2 transcripts were considered as negative.

### Measurement of Antibody Response in Serum

Antibody titers of IgG and IgA isotypes against CS5 and CS6 were determined as described previously [25] using purified CS5 (1 µg/ml) and recombinant CS6 (0.3 µg/ml) as coating antigens.

### Statistical Analyses

Results were analyzed with the non-parametrical non-paired Mann-Whitney test using the GraphPad Prism version 4.00 for Windows (GraphPad Software, San Diego, CA, USA).
